# Incorporating Transition-Affirming Language into Anatomical Pathology Reporting for Gender Affirmation Surgery

**DOI:** 10.1089/trgh.2019.0026

**Published:** 2019-11-21

**Authors:** Tehmina Ahmad, Anthea Lafreniere, David Grynspan

**Affiliations:** ^1^ Department of Medicine, University of Toronto, Faculty of Medicine, Toronto, Canada.; ^2^ Department of Pathology and Laboratory Medicine, University of Ottawa Faculty of Medicine, Ottawa, Canada.; ^3^ Department of Pathology and Laboratory Medicine, Vernon Jubilee Hospital, Vernon, Canada.

**Keywords:** gender transition, health screening, sex reassignment surgery, transgender

## Abstract

**Background:** The use of inclusive terminology in health records continues to be a challenge for transgender, gender-diverse, and nonbinary peoples. When patients access electronic health records, laboratory results, including pathology reports, are among the most frequently viewed items. There has been limited discussion of transgender care within laboratory medicine, despite its role in providing written pathology reports after gender-affirming surgery.

**Proposal:** This group proposes inclusive diagnostic terminology for pathology reporting and puts forward recommendations for procedural descriptions in the pathology report. Finally, we highlight pathological information that should be included in a report that has future cancer screening or diagnostic consequences.

In the North American health care setting, there is increasing recognition of the importance of providing inclusive comprehensive care to transgender, gender-diverse, and nonbinary (TGDNB) peoples.^1–3^ Transgender is a term used to describe individuals who experience their gender identity differently from the sex they were assigned at birth.^4^ Accurate representation of their gender identity within health records continues to be a challenge for TGDNB peoples as the health care community struggles to use inclusive terminology in medicine.^5^

It has been >25 years since the Canadian McInerney v. MacDonald (1992) case established that patients have the right to access information contained in their health records, and in the United States, the Health Insurance Portability and Accountability Act (HIPAA) Privacy Rule enfranchises those same rights.^6,7^ With ongoing health care advances, medical documentation has dramatically changed from hand-written notes to online documentation. These electronic medical records (EMRs) serve to digitally immortalize patient's health histories and health care.

Over the past two decades, health care in North America has moved toward a person-centered model. As a result of this movement, the availability of patient-accessible EMRs has increased considerably. Enhanced patient access to their personal health information supports shared decision-making, improves communication with health care providers, and allows for any concerns or inaccuracies to be corrected in the record—necessitating thoughtful consideration to inclusive medical language used within patient EMRs.^8,9^

Readily available EMR information removes the added barrier of formally requesting copies of health record reports and supports self-management by promoting health care knowledge and awareness.^10^ Access to health information drives patient convenience, strengthens patient–provider communication, and increases feelings of empowerment over one's agency of care.^11,12^ When patients access their EMRs, laboratory results, including pathology reports, are among the most frequently viewed items.^13,14^ In an oncological setting, for instance, the pathology report is an important source of information regarding diagnoses, treatment, and prognoses.

As part of a process referred to as gender transition, transgender people often seek medical care, including hormone therapy or surgery, to instigate changes in their secondary sex characteristics that more closely approximate the gender with which they self-identify.^5^ Gender-affirming surgical procedures include, but are not limited to, penectomy, orchiectomy, reduction thyrochondroplasty, total hysterectomy with bilateral salpingo-oophorectomy, and bilateral total reduction mammoplasty, all of which can result in surgical specimens requiring pathology reports.

Pathology reports have traditionally functioned as a communication tool solely between physicians; however, EMRs containing pathology results serve as an opportunity to optimize communication for transgender individuals, thereby alleviating the stress and stigma associated with viewing their health care record. Although these procedures rarely result in concerning pathological findings, the advent of patient-accessible pathology reports places a greater onus on the pathologist to use appropriate and objective language, particularly for transgender patients.

Within laboratory medicine, there has been limited discussion of its role in providing and optimizing transgender care. Gupta et al. described the lack of standardization of laboratory reference values in hematology and biochemistry and the difficulty in assessing screening tests, such as Pap smears, in transitioned individuals.^15^ They also remarked on the challenge of assessing biopsy specimens that may be influenced by exogenous hormones, such as prostate or breast tissue; however, they did not elaborate on the appropriate terminology for surgical sign out. Imborek et al. also noted the challenges of interpreting laboratory tests after transition; however, this group further explored challenges within laboratory medicine that comes from the incorporation of preferred name and gender identity, such as regulations regarding blood donor eligibility criteria in transfusion medicine and institutional barriers to specimen labeling with preferred names.^16^

It is well established that transgender patients often report acute discomfort when addressed according to a gender that is discordant with the gender with which they self-identify and it is for this reason that EMRs are increasingly incorporating gender identity data.^17–19^ Pathology reports that include gendered language such as “female breast tissue” rather than utilizing objective language such as “breast tissue with terminal duct lobular units” create a health care barrier in the form of disenfranchising the patient and adding to the lived reality of discrimination and harassment faced by trans individuals.^20^

To best serve transgender patients while maintaining a high standard of diagnostic integrity, we propose a set of inclusive terminology to be utilized in pathology reports for gender-affirming surgery (see Table 1). We outline terminology for tissue that has no histopathological abnormality as well as tissue that demonstrates changes associated with exogenous hormones. In Table 2, we further outline best practices in pathology reporting for transgender patients as well as clarifying pathology report items that should or should not be included within the pathology report.

**Table 1.** Suggested Terminology for Anatomical Pathology Reporting for Gender-Affirming Surgery

Specimen/procedure	Suggested diagnosis (No exogenous hormone exposure)	Suggested diagnosis (Exogenous hormone exposure)	Discouraged terminology
Orchiectomy	Benign testicular tissue or parenchyma	Benign testicular tissue with changes consistent with exogenous hormone exposure	Male testicular tissue
		Testicular atrophy	“Feminization” of testicular tissue
		Decreased spermatogenesis	
		Hypospermatogenesis	
		Maturation arrest	
		Germ cell aplasia	
Penectomy	Benign penile tissue or parenchyma	No findings expected	
Hysterectomy	Benign endomyometrium tissue or parenchyma	Benign endometrium with changes consistent with exogenous hormone exposure	
		Inactive endometrium	
Salpingectomy	Benign fallopian tube tissue or parenchyma	No findings expected	
Oophorectomy	Benign ovarian tissue or parenchyma	Benign ovarian tissue with changes consistent with exogenous hormone administration	
		Benign ovarian tissue with histological changes mimicking polycystic ovarian syndrome	
Mastectomy	Benign breast tissue	Benign breast tissue with changes consistent with exogenous hormone exposure	Female breast tissue
	Benign mammary tissue	Breast tissue with terminal duct units with lobular atrophy	Male breast tissue
	Benign fibroadipose tissue with terminal duct lobular units		Masculinization of breast tissue
			Gynecomastia (in the context of exogenous androgen exposure)

**Table 2.** Reporting of Gender-Affirming Surgical Procedures in the Anatomical Pathology

When describing the procedure, do include the technical name for the surgery.
Penectomy
Orchiectomy
Hysterectomy (total, subtotal/supracervical)
Oophorectomy
Salpingectomy
Mastectomy
When describing the procedure, as with all other pathology reporting, the reason for performing the surgery is not required. If, as a pathologist, you believe there is a medically necessary reason to include the rationale, the most appropriate term is “gender affirmation” or “gender-affirming” surgery.
In particular, the following terminology should be avoided in pathology reports:
Gender reassignment
Sex reassignment
Gender dysphoria treatment/reversal/therapy
Do highlight the presence or absence of tissue within the surgical specimen that may have future cancer screening or diagnostic consequences.^22–24^
Examples include:
Presence of exocervix in hysterectomy specimens
Completeness and bilaterality of fallopian tubes in salpingectomy specimens
Bilaterality of ovaries in oophorectomy specimens
While the prostate is seldom removed in gender affirmation surgery, its presence and completeness should be noted.
Any pathology present in a gender-affirming surgical specimen should be appropriately reported.

It will require infrastructural changes in EMRs and the institution of appropriate medical education to appropriately remove unnecessary gendered terms from pathology reports.^18^ Budding efforts toward integrating transgender health care training into medical curricula at the undergraduate and postgraduate levels are emerging but progress remains nascent.^21^ To further overcome transgender populations' experience of health inequities, additional training opportunities should be made available to practicing pathologists to help longitudinally integrate gender-neutral and nonexclusionary transgender language into EMRs through the surgical sign out. By keeping the specimen-naming and diagnostic line items objective, we further uphold the crux of professionalism and leadership in pathology, serve our transgender patients, and lead by example as the “Doctor's doctor.”

## Abbreviations Used

EMR: electronic medical record
HIPAA: Health Insurance Portability and Accountability Act
TGDNB: transgender, gender-diverse, and nonbinary
